# About the Treatment of Kasabach-Merritt Syndrome

**DOI:** 10.4274/tjh.02703

**Published:** 2013-03-05

**Authors:** Şinasi ÖZSOYLU

**Affiliations:** 1 Retired Professor of Pediatrics, Hematology, and Hepatology; 2 Honorary Fellow of American Academy of Pediatrics; 3 Honorary Member of American Pediatric Society; 4 Fellow of Islamic World Academy of Sciences

**To the Editor,**

Dr. Emre and colleagues briefly reported on a 24-year-old female with Kasabach-Merritt syndrome in the most recent issue of this journal [1].

Three units of fresh frozen plasma were administrated within 3 days to the patient for the correction of severe hypofibrinogenemia despite a very high D-dimer level (5000 ng/mL), both of which reflected consumption coagulopathy, as stated by the authors.

Although post-transfusion laboratory findings were not reported and consumption coagulopathy did not seem to be aggravated in the authors’ patient, I would like to draw attention to the complication of substrate supplementation in consumption coagulopathy cases without the taking of necessary precautions.

On such an occasion, I urge our approach of mega-dose methylprednisolone treatment (MDMP; daily, 30 mg/kg for 3 days, then 20 mg/kg for 4 days, and subsequently 10, 5, 2, and 1 mg/kg with each dose administered for 1 week, around 6 AM as a single dose). This was originally given intravenously, but the oral route is now recommended. Although both routes seem to be equally effective, oral administration is more practical, is cheaper, and does not require patient admission [2,3,4,5].

## REPLY

**Dear Editor,**

We have appreciated the valuable contributions of Prof.Dr. Şinasi ÖZSOYLU. Fresh frozen plasma (FFP) is among the options for treatment in KMS as well as steroid therapy. As intracranial haemorrhage was present in our case, FFP was preferred initially for the treatment of hypofibrinogenemia. As there was not any systemic complication during the clinical course; steroid treatment was not commenced during the follow-up. During the follow up, there has not been observed clinical complication associated with FFP treatment. We have not observed any complications during the FFP treatment during the follow-up. Certainly, it is imperative to pay utmost care in terms of KMS treatment algorithms and therapeutic complications because of the continuous presence of coagulopathic condition in these patients.

**With regards**

**Ufuk Emre**
